# Early Mortality in Reoperative Cardiac Surgery: Determinants, Prediction, and Calibration

**DOI:** 10.3390/diagnostics16152451

**Published:** 2026-08-03

**Authors:** Abdul Kerim Buğra, Tuba Mutu, Burak Ersoy, Zihni Mert Duman, Aytül Buğra

**Affiliations:** 1 Cardiovascular Surgery Department, Istanbul Mehmet Akif Ersoy Thoracic and Cardiovascular Surgery Training and Research Hospital, University of Health Sciences, 34303 Istanbul, Türkiye; mutu.tuba@gmail.com (T.M.); drburakersoy@gmail.com (B.E.); 2 Department of Cardiovascular Surgery, Faculty of Medicine, Pamukkale University, 20070 Denizli, Türkiye; dumanzihnimert@gmail.com; 3 Morgue Department, Council of Forensic Medicine, Ministry of Justice, 34196 Istanbul, Türkiye; aytulsargan@gmail.com

**Keywords:** reoperation, operative mortality, failure-to-rescue, endocarditis, low cardiac output, risk prediction, EuroSCORE II, cardiac surgery

## Abstract

**Background/Objectives:** Reoperative (redo) cardiac surgery carries elevated risk, yet determinants and mechanisms of early death in valve- and endocarditis-predominant cohorts are poorly described. We aimed to identify preoperative determinants of early mortality, derive a clinically usable risk model, examine the mechanisms of death, and evaluate EuroSCORE II. **Methods:** We analysed all redo cardiac operations through repeat sternotomy at a single tertiary centre (December 2010–December 2025); the cohort comprised 821 patients with verified in-hospital mortality status. Independent preoperative predictors were identified by multivariable logistic regression. Discrimination and calibration of the model and of EuroSCORE II were compared, and procedural complexity, graded low-cardiac-output severity, postoperative morbidity, and failure-to-rescue were examined. **Results:** The early mortality rate was 22.8% (187/821). The independent preoperative predictors were chronic kidney disease (adjusted odds ratio 2.25), age ≥70 years (2.32), active endocarditis (1.89), advanced functional class (1.78), non-elective status (1.69), and systolic pulmonary artery pressure (1.15 per 10 mmHg); the model discriminated and calibrated well (concordance statistic 0.755). EuroSCORE II discriminated similarly but systematically under-predicted mortality (observed-to-expected ratio 1.46; worst 2.06 at intermediate risk). Mortality rose with procedural complexity (17.3% to 37.3%) and graded haemodynamic support (3.4% to 79.1%). Failure-to-rescue was 38.5%, compared with 5.8% mortality in patients without a major complication. A standard-risk subgroup (*n* = 209) had 12.4% mortality. **Conclusions:** Early mortality after redo cardiac surgery is driven by identifiable preoperative comorbidity, procedural complexity, and low-cardiac-output physiology, and is concentrated in failure-to-rescue. A parsimonious preoperative model predicted mortality well; EuroSCORE II, although discriminating, systematically under-predicted risk, supporting redo-specific assessment and recalibration.

## 1. Introduction

Reoperative cardiac surgery is a growing share of the cardiac surgical workload, driven by an ageing population, wider use of degeneration-prone bioprostheses [1], and improved survival after index operations. Reoperation has historically carried greater morbidity and mortality than first-time surgery, owing to resternotomy, adhesions, and prior grafts, in a more comorbid and often more acute population [2,3,4].

Whether reoperation independently determines outcome or merely marks higher baseline risk has been debated. In a large propensity-matched analysis it remained an independent predictor of short- and long-term mortality after risk adjustment [5], suggesting an intrinsic hazard. Yet most contemporary scores, including EuroSCORE II, were developed in first-time populations [6], and external validations repeatedly show good discrimination but unreliable calibration in higher-risk or atypical cohorts [7,8,9,10,11]. Although EuroSCORE II already includes previous cardiac surgery among its risk factors, it may nonetheless mis-estimate risk in contemporary reoperative cohorts whose case-mix differs from the model’s derivation population.

Few preoperative tools are specific to redo surgery; the most cited is a single-centre scorecard stratifying redo patients into six categories from preoperative variables [12]. However, the literature is dominated by coronary-predominant Western cohorts; younger, valve- and endocarditis-heavy redo populations typical of tertiary referral practice outside high-income settings remain underrepresented, and their mechanisms of death are incompletely described.

We therefore analysed a contemporary, single-centre, all-comers redo cardiac surgery experience with three linked aims: to identify the preoperative determinants of early mortality and derive a parsimonious, clinically usable risk model; to examine the mechanisms of death across procedural complexity, low-cardiac-output severity, postoperative morbidity, and failure-to-rescue; and to test how well EuroSCORE II discriminates and calibrates in this population.

## 2. Materials and Methods

### 2.1. Study Design and Population

This retrospective analysis included all consecutive patients who underwent reoperative cardiac surgery through a repeat median sternotomy at the Department of Cardiovascular Surgery, Mehmet Akif Ersoy Thoracic and Cardiovascular Surgery Training and Research Hospital, between December 2010 and December 2025. Of 828 redo operations identified, 821 with verified in-hospital mortality status constituted the analytic cohort; 7 records were excluded as non-reoperative, not operated, or duplicate (Supplementary Figure S2). The study was approved by the hospital’s Scientific Research Ethics Committee (approval no. 2026.04-42, 14 April 2026); individual consent was waived given the retrospective design. Chronic kidney disease (CKD) was defined as a documented preoperative clinical diagnosis of chronic renal impairment; active endocarditis as endocarditis under treatment at the time of surgery. Baseline serum creatinine and estimated glomerular filtration rate were not available for retrospective KDIGO staging, so CKD could not be graded by laboratory criteria. Active endocarditis denoted a documented clinical diagnosis of active infective endocarditis at the index reoperation (modified Duke criteria as applied clinically), which itself constituted the reoperative indication; prior, treated (healed) endocarditis in patients reoperated for another indication was not separately recorded.

### 2.2. Outcomes and Definitions

The primary outcome was early (in-hospital) mortality. Preoperative variables comprised demographics, cardiovascular comorbidities, New York Heart Association (NYHA) functional class, left ventricular ejection fraction (LVEF), systolic pulmonary artery pressure (PAP), urgency, and active infective endocarditis; non-elective status denoted an urgent or emergent operation. EuroSCORE II was calculated for each patient. Postoperative morbidity was pregrouped as major cardiac/circulatory (mechanical circulatory support [intra-aortic balloon pump (IABP) or extracorporeal membrane oxygenation (ECMO)], early re-exploration, or new permanent pacemaker) and major non-cardiac (prolonged ventilation ≥3 days [≥72 h; ventilation was recorded in whole days, and this threshold was chosen to capture clinically significant prolonged ventilation while minimising misclassification at the 24–48 h boundary, with a secondary analysis using the conventional ≥48 h threshold], respiratory, gastrointestinal, mediastinitis, or wound infection). Failure-to-rescue was defined as mortality among patients sustaining at least one major complication. Early re-exploration was recorded as a binary event; specific indications (e.g., bleeding or suspected tamponade) were not systematically coded, so this category is heterogeneous. Because mechanical circulatory support contributed both to the low-cardiac-output severity axis and to the major-complication set, a sensitivity analysis recomputed failure-to-rescue after excluding mechanical support from the complication set.

### 2.3. Statistical Analysis

Continuous variables are reported as median [interquartile range] and compared with the Mann–Whitney U test; categorical variables are reported as *n* (%) and compared with the Fisher exact test. Candidate predictors were pre-specified on clinical grounds; variables of lesser a priori relevance (smoking, hypertension, atrial fibrillation, prior stroke) were summarised descriptively but not entered, to limit overfitting. The multivariable model used complete cases (*n* = 685, 152 deaths); with 11 degrees of freedom the events-per-variable ratio was 13.8. Patients excluded for a missing covariate (*n* = 136, predominantly unrecorded systolic PAP) did not differ in early mortality (25.7% versus 22.2%; *p* = 0.37), supporting a complete-case approach. As a sensitivity analysis, missing preoperative covariates (principally systolic PAP) were additionally handled by multiple imputation by chained equations, with the model refitted in the full cohort (*n* = 821) and compared with the complete-case model (Supplementary Table S6). Age violated logit linearity (Box–Tidwell *p* = 0.01) and was modelled categorically (<60, 60–70, ≥70 years); LVEF and PAP satisfied linearity and were retained as continuous per-10-unit terms. LVEF was kept a priori despite falling short of significance (adjusted *p* = 0.15), given its established prognostic role, protective direction, and contribution to calibration. Multicollinearity was excluded (variance inflation factors ≈ 1.1). Discrimination (C-statistic) and calibration were assessed, with internal validity by bootstrap resampling (1000 samples) with optimism correction [13]. Calibration was examined by the Hosmer–Lemeshow test, a calibration plot by risk decile, and the calibration slope and intercept [14]. EuroSCORE II discrimination and calibration (observed/expected, overall and by predicted-risk band) were compared with the model, with overall accuracy summarised by the Brier score and calibration-in-the-large. A pre-specified sensitivity analysis compared profile and early mortality between earlier (2010–2017) and later (2018–2025) eras. Intraoperative markers were analysed descriptively only. Analyses used R version 4.3.2 (R Foundation for Statistical Computing, Vienna, Austria). Two-sided *p* < 0.05 defined significance.

## 3. Results

### 3.1. Cohort and Baseline Characteristics

Among 821 patients, 187 died in hospital (early mortality 22.8%). The median age was 56 [46–65] years and 52.3% were female. Compared with survivors, non-survivors had higher rates of diabetes, CKD, chronic obstructive pulmonary disease (COPD), prior myocardial infarction, NYHA III–IV, non-elective presentation, and active endocarditis, with lower LVEF and higher systolic PAP and EuroSCORE II. Intraoperatively, non-survivors had femoral cannulation and circulatory arrest more often and longer cardiopulmonary bypass (CPB) and cross-clamp times (Table 1). Early mortality did not differ significantly by sex (men 20.7%, women 24.7%; *p* = 0.18); characteristics stratified by sex are presented in Supplementary Table S3.

### 3.2. Preoperative Predictors and Risk Model

**Figure 1:**
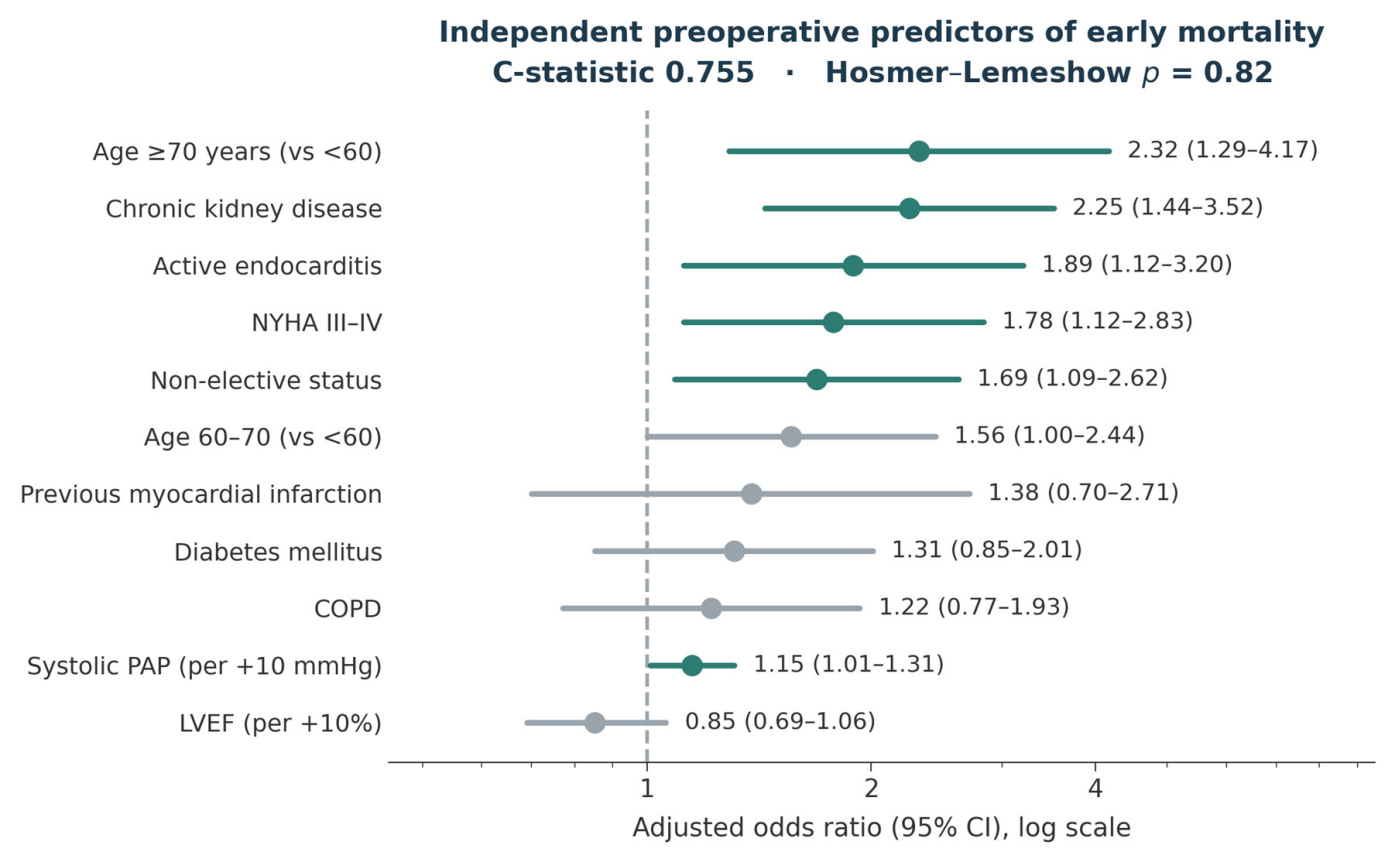
Adjusted odds ratios (95% confidence intervals) for the independent preoperative predictors of early mortality; significant predictors are highlighted. Numerical estimates are given in Supplementary Table S1. Model performance is shown in the figure (C-statistic 0.755; Hosmer–Lemeshow *p* = 0.82); the odds-ratio axis is on a logarithmic scale.

Six factors were independently associated with early mortality: CKD (adjusted odds ratio [aOR] 2.25), age ≥ 70 years (2.32), active endocarditis (1.89), NYHA III–IV (1.78), non-elective status (1.69), and systolic PAP (1.15 per +10 mmHg); higher LVEF showed a protective trend (0.85 per +10%) (Figure 1; Supplementary Table S1). The model discriminated well (C-statistic 0.755, bootstrap 95% CI 0.714–0.796; optimism-corrected 0.732) and was well calibrated (Hosmer–Lemeshow *p* = 0.82; optimism-corrected calibration slope 0.88, intercept ≈ 0; Brier score 0.149; calibration-in-the-large ≈ 0) (Supplementary Figure S1); the events-per-variable ratio was 13.8. Case-mix did not differ between the 2010–2017 and 2018–2025 eras (all *p* > 0.05) and early mortality was similar (24.6% vs. 22.5%), arguing against a learning-curve or historical-bias effect. In a multiply imputed sensitivity analysis (*n* = 821), all independent predictors retained their direction and magnitude and discrimination was preserved (Supplementary Table S6), indicating that exclusion of cases with missing pulmonary artery pressure did not bias the model.

### 3.3. Comparison with EuroSCORE II

**Figure 2:**
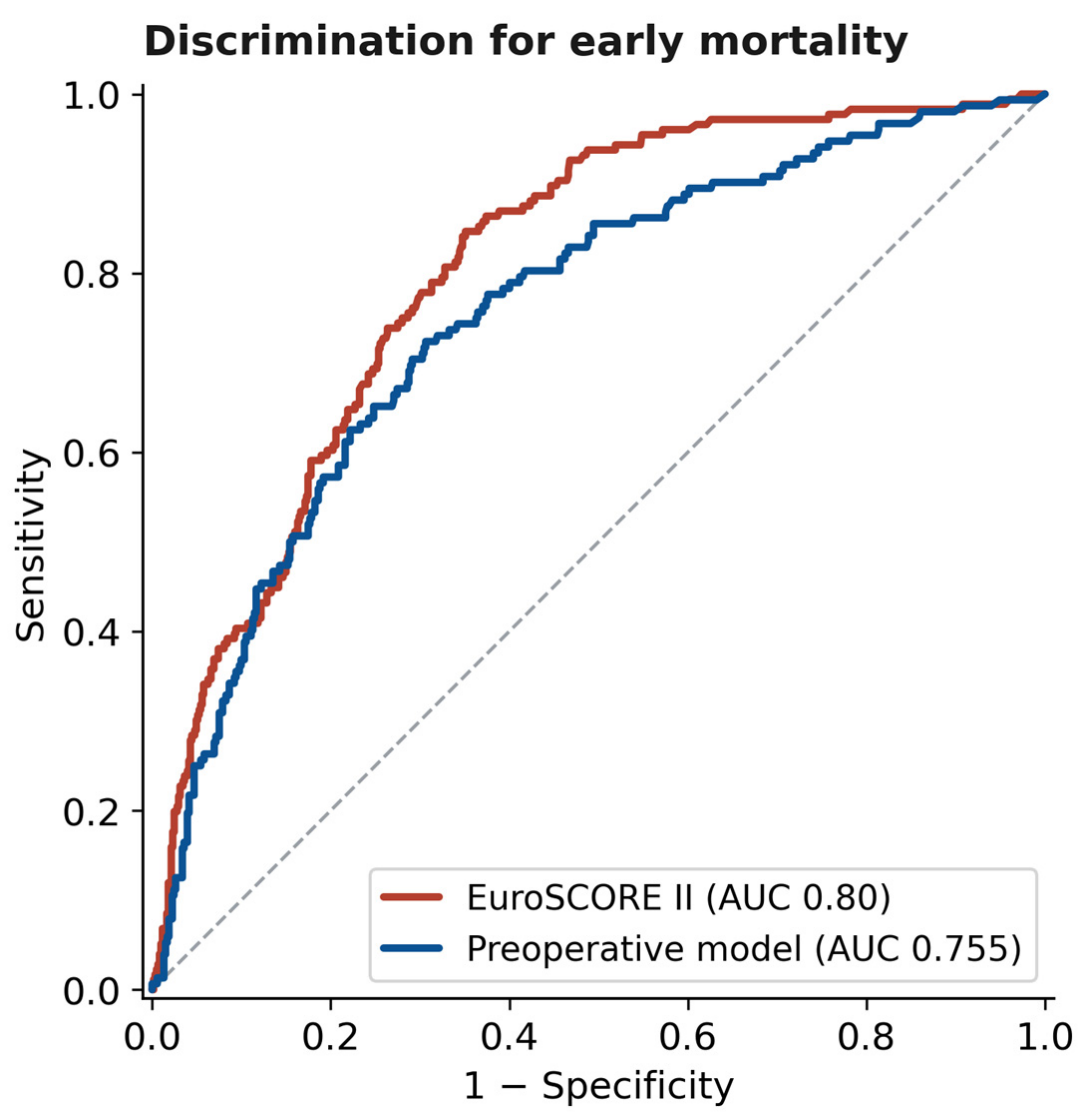
Receiver-operating-characteristic curves for early mortality: EuroSCORE II versus the preoperative model (area under the curve 0.80 versus 0.755).

**Figure 3:**
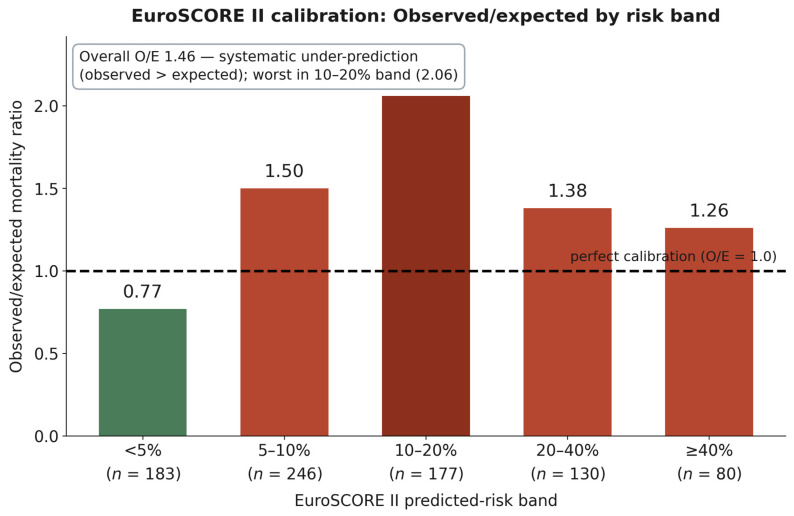
EuroSCORE II calibration as the observed/expected mortality ratio by predicted-risk band (dashed line, perfect calibration). The overall observed/expected ratio was 1.46, greatest in the 10–20% band (2.06).

EuroSCORE II discriminated slightly better than the preoperative model (area under the curve 0.80 vs. 0.755; Figure 2) but was poorly calibrated, systematically under-predicting observed mortality (observed/expected 1.46). Miscalibration was greatest in the intermediate-to-high range, with an observed/expected of 2.06 in the 10–20% predicted band (observed mortality roughly twice that predicted; Figure 3). Comparable under-prediction or unreliable calibration of EuroSCORE II in high-acuity, reoperative, and octogenarian cohorts has been widely reported [7,10,11,15,16]. In other words, EuroSCORE II rank-orders reoperative patients well but lacks calibration-in-the-large.

### 3.4. Procedural Complexity and Low-Cardiac-Output Severity

**Figure 4:**
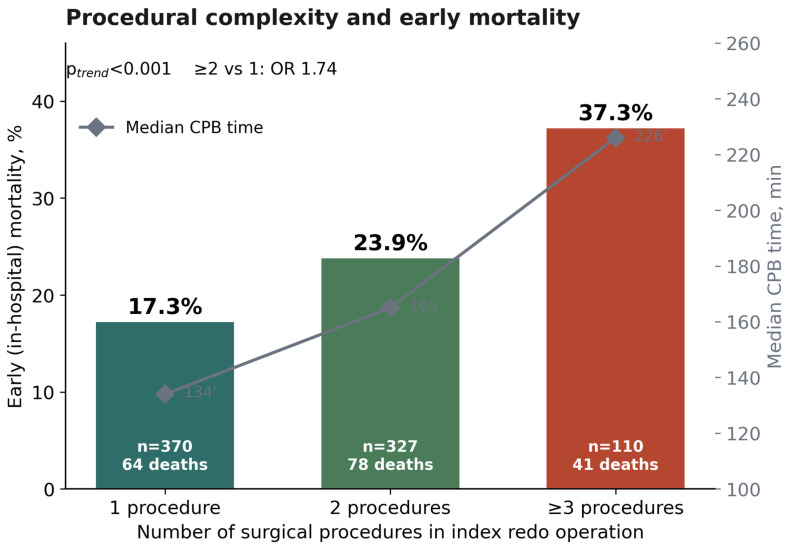
Early mortality by number of concomitant procedures, overlaid with median cardiopulmonary bypass time.

**Figure 5:**
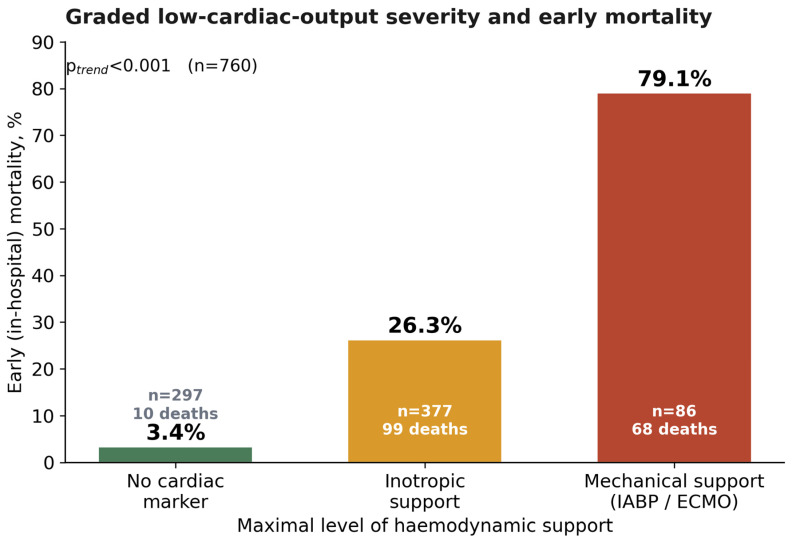
Early mortality across graded low-cardiac-output severity (no marker, inotropic support, and mechanical support [intra-aortic balloon pump or extracorporeal membrane oxygenation]).

Early mortality increased monotonically with the number of concomitant procedures (17.3%, 23.9%, 37.3% for 1, 2, ≥3 procedures; *p* for trend < 0.001), and combined (≥2-procedure) operations carried higher risk than single-procedure operations (OR 1.74, 95% CI 1.24–2.43). The gradient tracked a parallel rise in median CPB time (134, 165, 226 min), suggesting a mechanistic relationship (Figure 4). Mortality was further concentrated along the low-cardiac-output axis: 3.4% with no haemodynamic support marker, 26.3% with inotropic support, and 79.1% with mechanical circulatory support (IABP or ECMO) (*p* for trend < 0.001) (Figure 5).

### 3.5. Postoperative Morbidity and Failure-to-Rescue

Postoperative morbidity was frequent and strongly linked to death. Major cardiac/circulatory complications occurred in 259 patients (31.5%, 48.3% mortality) and major non-cardiac complications in 347 (42.3%, 39.5% mortality); the steepest gradients were mechanical support (79.1%) and early re-exploration (53.8%) (Table 2). New-onset postoperative atrial fibrillation occurred in 306 patients (37.3%, 26.5% mortality). A major complication occurred in 51.9% of the cohort, and mortality among these patients (failure-to-rescue) was 38.5%, compared with 5.8% in those without a major complication, indicating that death clustered in patients who could not be rescued rather than those who simply developed a complication [17,18,19]. The morbidity spectrum mirrors multicentre redo series [20]. Prolonged ventilation was associated with markedly higher mortality at both thresholds (≥72 h: 48.0% versus 12.5%; ≥48 h: 42.7% versus 10.1%). Excluding mechanical circulatory support from the complication set, failure-to-rescue was essentially unchanged (38.1% versus 38.5%), confirming that the finding was not an artefact of classifying terminal salvage support as a complication.

### 3.6. Risk Strata and the Standard-Risk Subgroup

Literature-aligned strata reproduced expected gradients [20,21]: combined (≥2) procedures (OR 1.74), bypass time ≥ 180 min (OR 2.91), and NYHA III–IV (OR 2.62) were each strongly associated with mortality, which rose across age bands (15.9%, 27.9%, 43.6% for <60, 60–69, 70–79 years). Isolated redo of the same structure was not significant (OR 0.69, 95% CI 0.43–1.10). A standard-risk subgroup (elective, single procedure, no active endocarditis; *n* = 209) had an early mortality of 12.4% (Supplementary Table S2).

## 4. Discussion

In this contemporary, all-comers redo cohort, early mortality was 22.8%, driven by identifiable preoperative comorbidity, procedural complexity, and low-cardiac-output physiology, and concentrated in failure-to-rescue. A parsimonious preoperative model predicted mortality well (C-statistic 0.755) and was well calibrated, whereas EuroSCORE II discriminated comparably but systematically under-predicted risk. Early death therefore reflected patient and procedural risk, together with the ability to rescue patients, more than any single score.

The overall mortality warrants context. Our cohort was younger (median 56 years) and dominated by valve and infective-endocarditis indications, with many outside-hospital referrals (66.5%), a case-mix that concentrates high-acuity physiology, with the highest mortality in active endocarditis, non-elective, and high-complexity operations (Supplementary Table S4). Restricted to a standard-risk subgroup (elective, single-procedure, endocarditis-free), mortality fell to 12.4%, approaching but still above the published redo range of roughly 4.6% to 9.7% [12,22] and 8.37% in a propensity-matched North American experience [5], with a multicentre redo-mitral registry showing a comparable high-risk profile [20]. Even here, observed mortality was about twice the median EuroSCORE II prediction (5.5%), indicating an excess driven by the intrinsic risk of reoperation beyond case-mix alone, though unmeasured centre-level factors cannot be excluded.

EuroSCORE II behaved as expected for a score developed largely in first-time surgery: adequate discrimination but poor absolute accuracy, with under-prediction worst in the intermediate-to-high range. Although a tertiary cohort enriched for high-acuity referrals will somewhat exceed population-derived predictions, the size and risk-dependent pattern of under-prediction indicate genuine miscalibration rather than a case-mix shift. Similar findings recur across external validations and high-acuity or octogenarian cohorts [7,8,10,11,15,16]. Because clinical decisions depend on absolute risk, this miscalibration supports redo-specific recalibration rather than off-the-shelf application of the model; our model may serve as a decision-support framework, pending external validation.

The independent predictors are clinically coherent. The non-linear age effect, with risk accelerating beyond 60 years, reproduces the incremental age gradient in the redo scorecard literature [12] and argues against a universal per-year assumption. Active endocarditis remained independently predictive (aOR 1.89), modelled as a standalone variable rather than folded into a composite urgency index. CKD was the strongest comorbidity (aOR 2.25), with non-elective status and advanced functional class retaining independent weight.

Mortality was mechanistically graded: the monotonic rise with complexity tracked bypass time [21], and the steep low-cardiac-output gradient localised death to the low-output axis. Although roughly half the cohort sustained a major complication, mortality among them (38.5%) far exceeded that of complication-free patients (5.8%), with the steepest gradients in mechanical support and re-exploration. Survival therefore depends less on preventing every complication than on rescuing patients once low-output physiology or bleeding supervenes [2,3,4]; that reoperation remains an independent predictor even after propensity matching [5] is directly relevant to decisions between reoperation and transcatheter alternatives [1,2,4].

Unlike redo series identifying postoperative predictors such as new-onset atrial fibrillation [22], our model uses only preoperative variables and is usable before the decision to operate. Intraoperative markers (cannulation, circulatory arrest, bypass time), though strongly associated with death, are confounded by indication and unavailable preoperatively; they are reported descriptively (Table 1) rather than used for prediction.

The infective subset deserves specific comment. Active endocarditis (12.5% of the cohort) remained independently associated with early death (OR 1.89), consistent with contemporary endocarditis surgery, where operative mortality is high and driven by similar factors. In a high-risk surgical endocarditis series, Nasso and colleagues reported 27% mortality among patients with a prior aortic valve replacement and higher mortality during the active phase, with renal failure, raised EuroSCORE II, and prolonged cross-clamp time as dominant predictors [23]; a meta-analysis likewise identified renal dysfunction, heart failure, and prosthetic involvement as the most consistent predictors [24]. This overlap with our leading determinants places the reoperative infective patient at the intersection of two high-risk phenotypes. Active endocarditis was also closely tied to acuity in our cohort, with the majority of these patients operated non-electively.

The mechanistic pattern aligns with the wider literature. Prolonged CPB and a low-output state were the proximate correlates of death across complexity strata, echoing the cross-clamp-time relationship in endocarditis surgery [23] and the low-output and prolonged-ventilation events that dominate redo series, including the redo-mitral experience, where balloon pumping, prolonged intubation, and myocardial infarction independently predicted operative death [25]. That mortality concentrated in failure-to-rescue rather than the occurrence of a complication accords with the failure-to-rescue concept [17,18,19], shifting the clinical target from complication avoidance, which is often unrealistic in scarred, infected, or reoperated fields, towards earlier recognition and aggressive treatment of low-output physiology and a low threshold for proactive mechanical circulatory support before shock is established.

These observations carry practical implications. Because EuroSCORE II under-predicted risk most in the intermediate band, reliance on it alone may falsely reassure the heart team when counselling reoperative candidates, consistent with a recent validation in emergency and high-risk patients [26]; a redo-specific, recalibrated estimate is preferable for shared decision-making. In selected patients with a degenerated bioprosthesis, transcatheter valve-in-valve treatment may avoid a high-risk resternotomy [1,2,4,27], and a reliable preoperative estimate may identify those who benefit most. Where reoperation is unavoidable, the data support concentrating patients in experienced teams with a structured rescue pathway [28]. External validation is needed before applying the model beyond our institution.

### Limitations

This single-centre retrospective study has the usual constraints, including selection bias and limited generalisability, although the tertiary referral pattern reflects a realistic redo case-mix. Low-cardiac-output severity was anchored on mechanical support because inotrope use was recorded only as a binary variable; systemic sepsis was not separately coded. Because mechanical support marks both the most severe low-output grade and a complication, that gradient and failure-to-rescue partly describe the same high-risk patients. CKD was captured as a documented clinical diagnosis rather than by creatinine, estimated glomerular filtration rate, or dialysis status, which were not uniformly recorded, precluding finer renal staging. Intraoperative markers, confounded by indication, were not used for prediction. Late survival was not analysed, as the study was restricted by design to early in-hospital outcomes; the recovery course of early survivors from this cohort is reported separately in a companion manuscript. Only pre-specified, clinically grounded predictors entered the model. This CKD definition introduces measurement bias, since mild renal impairment may be classified identically to dialysis dependence, limiting the granularity of the predictor and the portability of the model to centres using laboratory-based definitions. A sensitivity analysis excluding mechanical support from the complication set left failure-to-rescue essentially unchanged (38.1%), indicating that the finding is not merely an artefact of that overlap. Re-exploration was recorded as a binary event without systematically coded indications, and the active versus quiescent phase of endocarditis and prior treated endocarditis were not separately captured.

## 5. Conclusions

In a contemporary, younger, valve- and endocarditis-predominant redo cohort, early mortality of 22.8% may partly reflect a high-acuity case-mix, with a standard-risk subgroup mortality of 12.4%. Early death was driven by preoperative comorbidity, procedural complexity, and low-cardiac-output physiology, and concentrated in failure-to-rescue. A parsimonious preoperative model predicted mortality well, whereas EuroSCORE II under-predicted risk despite adequate discrimination, supporting redo-specific assessment and recalibration of generic scores. External, multicentre validation is the essential next step.

## Figures and Tables

**Table 1:** Baseline preoperative and operative characteristics of the cohort, overall and by early (in-hospital) mortality.

Variable	Overall (n = 821)	Non-Survivors (n = 187)	Survivors (n = 634)	p Value
**Preoperative Characteristics**				
Age, years	56 [46–65]	62 [51–69]	55 [44–63]	<0.001
Female sex	429 (52.3)	106 (56.7)	323 (50.9)	0.18
Diabetes mellitus	218 (26.6)	71 (38.0)	147 (23.2)	<0.001
Smoking	311 (37.9)	84 (44.9)	227 (35.8)	0.03
COPD	161 (19.6)	49 (26.2)	112 (17.7)	0.01
Chronic kidney disease	166 (20.2)	72 (38.5)	94 (14.8)	<0.001
Previous myocardial infarction	77 (9.4)	30 (16.0)	47 (7.4)	0.001
LVEF, %	55 [50–60]	50 [43–60]	55 [50–60]	<0.001
Systolic PAP, mmHg	38 [30–48]	40 [30–53]	37 [30–45]	<0.001
NYHA class III–IV	154 (18.8)	59 (31.6)	95 (15.0)	<0.001
Non-elective priority	221 (26.9)	68 (36.4)	153 (24.1)	0.001
Active endocarditis	103 (12.5)	37 (19.8)	66 (10.4)	0.002
Interval since last operation, years	8 [2–16]	8 [2–14]	8 [2–17]	0.10
Previous CABG	157 (19.1)	56 (29.9)	101 (15.9)	<0.001
Previous valve surgery	553 (67.4)	120 (64.2)	433 (68.3)	0.29
EuroSCORE II, %	9 [5–21]	22 [12–42]	8 [5–14]	<0.001
**Intraoperative (Descriptive)**				
Femoral/peripheral cannulation	282 (34.3)	84 (44.9)	198 (31.2)	0.001
Circulatory arrest	14 (1.7)	12 (6.4)	2 (0.3)	<0.001
CPB time, min	154 [113–205]	196 [143–260]	147 [109–190]	<0.001
Cross-clamp time, min	91 [69–133]	111 [78–158]	89 [66–123]	<0.001

Continuous data are median [interquartile range]; categorical data *n* (%). *p* from Mann–Whitney U or Fisher exact tests. Non-elective status denotes any urgent or emergent operation. Full baseline table (including hypertension, atrial fibrillation, prior stroke, referral source, pulmonary-hypertension bands, and prior-redo count) is provided in Supplementary Table S5. Abbreviations: CABG, coronary artery bypass grafting; COPD, chronic obstructive pulmonary disease; CPB, cardiopulmonary bypass; LVEF, left ventricular ejection fraction; NYHA, New York Heart Association; PAP, pulmonary artery pressure.

**Table 2:** Postoperative major complications and associated in-hospital mortality, including failure-to-rescue.

Complication	n (%)	In-Hospital Mortality, n (%)
**Major Cardiac/Circulatory**		
Mechanical support (IABP/ECMO)	86 (10.5)	68 (79.1)
Early re-exploration	195 (23.8)	105 (53.8)
New permanent pacemaker	62 (7.6)	17 (27.4)
Any major cardiac	259 (31.5)	125 (48.3)
**Major Non-Cardiac**		
Prolonged ventilation ≥3 days	244 (29.7)	117 (48.0)
Respiratory complication	255 (31.1)	107 (42.0)
Gastrointestinal complication	29 (3.5)	11 (37.9)
Mediastinitis	7 (0.9)	4 (57.1)
Wound infection	51 (6.2)	12 (23.5)
Any major non-cardiac	347 (42.3)	137 (39.5)
**Overall (Failure-to-Rescue)**		
Any major complication	426 (51.9)	164 (38.5)
No major complication	395 (48.1)	23 (5.8)

Percentages of cohort use *n* = 821; mortality percentages are within each exposed group. Abbreviations: ECMO, extracorporeal membrane oxygenation; IABP, intra-aortic balloon pump.

## Data Availability

The data presented in this study are available on request from the corresponding author. The data are not publicly available due to privacy and institutional restrictions.

## Supplementary Materials

The following supporting information can be downloaded at: https://www.mdpi.com/article/10.3390/diagnostics16152451/s1.

## References

[1] Goldstone A.B., Chiu P., Baiocchi M., Lingala B., Patrick W.L., Fischbein M.P., Woo Y.J. (2017). Mechanical or biologic prostheses for aortic-valve and mitral-valve replacement. N. Engl. J. Med..

[2] Imran Hamid U., Digney R., Soo L., Leung S., Graham A.N. (2015). Incidence and outcome of re-entry injury in redo cardiac surgery: Benefits of preoperative planning. Eur. J. Cardiothorac. Surg..

[3] Park C.B., Suri R.M., Burkhart H.M., Greason K.L., Dearani J.A., Schaff H.V., Sundt T.M. (2010). Identifying patients at particular risk of injury during repeat sternotomy: Analysis of 2555 cardiac reoperations. J. Thorac. Cardiovasc. Surg..

[4] Roselli E.E., Pettersson G.B., Blackstone E.H., Brizzio M.E., Houghtaling P.L., Hauck R., Burke J.M., Lytle B.W. (2008). Adverse events during reoperative cardiac surgery: Frequency, characterization, and rescue. J. Thorac. Cardiovasc. Surg..

[5] Bianco V., Kilic A., Gleason T.G., Aranda-Michel E., Habertheuer A., Wang Y., Navid F., Kacin A., Sultan I. (2020). Reoperative cardiac surgery is a risk factor for long-term mortality. Ann. Thorac. Surg..

[6] Nashef S.A., Roques F., Sharples L.D., Nilsson J., Smith C., Goldstone A.R., Lockowandt U. (2012). EuroSCORE II. Eur. J. Cardiothorac. Surg..

[7] Di Dedda U., Pelissero G., Agnelli B., De Vincentiis C., Castelvecchio S., Ranucci M. (2013). Accuracy, calibration and clinical performance of the new EuroSCORE II risk stratification system. Eur. J. Cardiothorac. Surg..

[8] Chalmers J., Pullan M., Fabri B., McShane J., Shaw M., Mediratta N., Poullis M. (2013). Validation of EuroSCORE II in a modern cohort of patients undergoing cardiac surgery. Eur. J. Cardiothorac. Surg..

[9] Carnero-AlcÁZar M., Guisasola J.A.S., Lacruz F.J.R., Castellanos L.C.M., Carnicer J.C., Medinilla E.V., SÁNchez T.T., HernÁNdez J.E.R. (2013). Validation of EuroSCORE II on a single-center 3800 patient cohort. Interact. Cardiovasc. Thorac. Surg..

[10] Grant S.W., Hickey G.L., Dimarakis I., Trivedi U., Bryan A., Treasure T., Cooper G., Pagano D., Buchan I., Bridgewater B. (2012). How does EuroSCORE II perform in UK cardiac surgery? An analysis of 23,740 patients. Heart.

[11] Barili F., Pacini D., Capo A., Rasovic O., Grossi C., Alamanni F., Di Bartolomeo R., Parolari A. (2013). Does EuroSCORE II perform better than its original versions? A multicenter validation study. Eur. Heart J..

[12] Launcelott S., Ouzounian M., Buth K.J., Légaré J.F. (2012). Predicting in-hospital mortality after redo cardiac operations: Development of a preoperative scorecard. Ann. Thorac. Surg..

[13] Steyerberg E.W., Vickers A.J., Cook N.R., Gerds T., Gonen M., Obuchowski N., Pencina M.J., Kattan M.W. (2010). Assessing the performance of prediction models: A framework for traditional and novel measures. Epidemiology.

[14] Van Calster B., McLernon D.J., van Smeden M., Wynants L., Steyerberg E.W. (2019). Calibration: The Achilles heel of predictive analytics. BMC Med..

[15] Kuplay H., Bayer Erdoğan S., Baştopçu M., Karpuzoğlu E., Er H. (2021). Performance of the EuroSCORE II and the STS score for cardiac surgery in octogenarians. Turk. Gogus Kalp Damar Cerrahisi Derg..

[16] Takkenberg J.J.M., Kappetein A.P., Steyerberg E.W. (2013). The role of EuroSCORE II in 21st century cardiac surgery practice. Eur. J. Cardiothorac. Surg..

[17] Silber J.H., Williams S.V., Krakauer H., Schwartz J.S. (1992). Hospital and patient characteristics associated with death after surgery: A study of adverse occurrence and failure to rescue. Med. Care.

[18] Ghaferi A.A., Birkmeyer J.D., Dimick J.B. (2009). Variation in hospital mortality associated with inpatient surgery. N. Engl. J. Med..

[19] Gonzalez A.A., Dimick J.B., Birkmeyer J.D., Ghaferi A.A. (2014). Understanding the volume-outcome effect in cardiovascular surgery: The role of failure to rescue. JAMA Surg..

[20] Onorati F., Mariscalco G., Reichart D., Perrotti A., Gatti G., De Feo M., Rubino A., Santarpino G., Biancari F., Detter C. (2018). Hospital outcome and risk indices of mortality after redo-mitral valve surgery in potential candidates for transcatheter procedures: Results from a European registry. J. Cardiothorac. Vasc. Anesth..

[21] Di Mauro M., Iacò A.L., Contini M., Teodori G., Vitolla G., Pano M., Di Giammarco G., Calafiore A.M. (2005). Reoperative coronary artery bypass grafting: Analysis of early and late outcomes. Ann. Thorac. Surg..

[22] Colkesen Y., Coskun I., Cayli M., Gulcan O. (2015). Predictors of in-hospital mortality following redo cardiac surgery: Single center experience. Interv. Med. Appl. Sci..

[23] Nasso G., Santarpino G., Moscarelli M., Condello I., Dell’aQuila A.M., Peivandi A.D., Gaudino M., Fiore F., Mastroroberto P., Di Bari N. (2021). Surgical treatment of valve endocarditis in high-risk patients and predictors of long-term outcomes. Sci. Rep..

[24] Barca L.V., Elorza E.N., Fernández-Hidalgo N., Mur J.L.M., García A.M., Fernández-Felix B.M., Hycka J.M., Rodríguez-Roda J., López-Menéndez J. (2019). Prognostic factors of mortality after surgery in infective endocarditis: Systematic review and meta-analysis. Infection.

[25] Onorati F., Perrotti A., Reichart D., Mariscalco G., Della Ratta E., Santarpino G., Salsano A., Rubino A., Biancari F., Gatti G. (2016). Surgical factors and complications affecting hospital outcome in redo mitral surgery: Insights from a multicenter experience. Eur. J. Cardiothorac. Surg..

[26] Mastroiacovo G., Bonomi A., Ludergnani M., Franchi M., Maragna R., Pirola S., Baggiano A., Caglio A., Pontone G., Polvani G. (2023). Is EuroSCORE II still a reliable predictor for cardiac surgery mortality in 2022? A retrospective study. Eur. J. Cardiothorac. Surg..

[27] Webb J.G., Mack M.J., White J.M., Dvir D., Blanke P., Herrmann H.C., Leipsic J., Kodali S.K., Makkar R., Miller D.C. (2017). Transcatheter aortic valve implantation within degenerated aortic surgical bioprostheses: PARTNER 2 valve-in-valve registry. J. Am. Coll. Cardiol..

[28] LaPar D.J., Ailawadi G., Harris D.A., Hajzus V.A., Lau C.L., Kern J.A., Kron I.L. (2013). A protocol-driven approach to cardiac reoperation reduces mortality and cardiac injury at the time of resternotomy. Ann. Thorac. Surg..

