# Supplementary material for: Early Mortality in Reoperative Cardiac Surgery: Determinants, Prediction, and Calibration
Source: Diagnostics (Basel). 2026 Aug 3;16(15):2451. doi: 10.3390/diagnostics16152451 (PMC13465322; doi:10.3390/diagnostics16152451)
Supplement: Supplementary file 1 [file diagnostics-16-02451-s001.zip › diagnostics-4439374-supplementary.pdf]

Supplementary Materials

Early Mortality in Reoperative Cardiac Surgery: Determinants, Prediction, and Calibration

Abdul Kerim Buğra, Tuba Mutu, Burak Ersoy, Zihni Mert Duman, Aytül Buğra

This file contains: Table S1, Table S2, Table S3, Table S4, Table S5, Table S6, Figure S1, Figure S2.

Table S1. Multivariable logistic regression model for early mortality (adjusted odds ratios).

Predictor	β	Adjusted OR	95% CI	P
Intercept	−2.12	–	–	–
Age 60–70 (vs <60)	0.445	1.56	1.00–2.44	0.050
Age ≥70 (vs <60)	0.842	2.32	1.29–4.17	0.005
LVEF, per +10%	−0.163	0.85	0.69–1.06	0.145
Systolic PAP, per +10 mmHg	0.140	1.15	1.01–1.31	0.030
Diabetes mellitus	0.270	1.31	0.85–2.01	0.224
COPD	0.199	1.22	0.77–1.93	0.391
Previous myocardial infarction	0.322	1.38	0.70–2.71	0.356
Chronic kidney disease	0.811	2.25	1.44–3.52	<0.001
Non-elective status	0.525	1.69	1.09–2.62	0.020
NYHA class III–IV	0.577	1.78	1.12–2.83	0.015
Active endocarditis	0.637	1.89	1.12–3.20	0.017

β values are logistic-regression log-odds coefficients; the linear predictor is logit(p) = −2.12 + Σ(β × variable), with age modelled categorically (reference <60 years) and LVEF and systolic PAP entered per 10-unit increment. Adjusted OR = exp(β). n=685; C-statistic 0.755 (optimism-corrected 0.732); calibration slope 0.88, intercept ≈0; Hosmer–Lemeshow p = 0.82. Abbreviations: CI, confidence interval; COPD, chronic obstructive pulmonary disease; LVEF, left ventricular ejection fraction; NYHA, New York Heart Association; OR, odds ratio; PAP, pulmonary artery pressure.

Table S2. Early mortality across pre-specified clinical strata.

Stratum	n	Deaths	Mortality %	OR (95% CI), P
**Procedural complexity (number of procedures)**				
Single-procedure	370	64	17.3	Reference
Combined (≥2)	437	119	27.2	1.74 (1.24–2.43), 0.001
**Cardiopulmonary bypass time**				
<180 min	495	75	15.2	Reference
≥180 min	272	98	36.0	2.91 (2.08–4.07), <0.001
**NYHA functional class**				
NYHA I–II	231	40	17.3	Reference
NYHA III–IV	154	59	38.3	2.62 (1.79–3.82), <0.001
**Age band**				

Stratum	n	Deaths	Mortality %	OR (95% CI), P
<60 years	479	76	15.9	Reference
60–69 years	244	68	27.9	–
70–79 years	94	41	43.6	–
**Isolated redo of the same structure**				
Isolated	136	24	17.6	Reference
Combined / new indication	685	163	23.8	0.69 (0.43–1.10), 0.120

Univariate strata; reference categories as indicated. Abbreviations: CI, confidence interval; NYHA, New York Heart Association; OR, odds ratio.

Table S3. Baseline characteristics and early mortality stratified by sex.

Variable	Male (*n*=392)	Female (*n*=429)	P
Early mortality, *n* (%)	81 (20.7)	106 (24.7)	0.183
Age, years	55 [44–63]	58 [47–66]	0.002
Diabetes mellitus, *n* (%)	103 (26.3)	115 (26.8)	0.875
Chronic kidney disease, *n* (%)	61 (15.6)	105 (24.5)	0.002
COPD, *n* (%)	58 (14.8)	103 (24.0)	0.001
LVEF, %	55 [50–60]	55 [45–60]	<0.001
Active endocarditis, *n* (%)	45 (11.5)	58 (13.5)	0.400
Elective priority, *n* (%)	268 (68.4)	297 (69.2)	0.821
EuroSCORE II, %	9 [6–17]	10 [5–26]	0.104
CPB time, min	147 [108–191]	166 [120–224]	<0.001

Continuous data are median [interquartile range]; categorical data n (%). p from Mann–Whitney U or Fisher exact tests. Abbreviations: COPD, chronic obstructive pulmonary disease; CPB, cardiopulmonary bypass; LVEF, left ventricular ejection fraction.

Table S4. Early mortality by operative priority, principal indication, and procedural complexity.

Category	n	Deaths	Mortality %
**Operative priority**			
Elective	565	110	19.5
Non-elective	221	68	30.8
**Principal indication (non-exclusive)**			
Aortic valve replacement	288	66	22.9
Mitral valve replacement	480	120	25.0
Tricuspid valve replacement	73	22	30.1
Aortic root / Bentall	55	15	27.3
Redo coronary bypass	102	30	29.4
Active infective endocarditis	103	37	35.9

Category	n	Deaths	Mortality %
**Procedural complexity**			
Single procedure	370	64	17.3
Two procedures	327	78	23.9
≥3 procedures	110	41	37.3

Indication categories are non-exclusive (a patient may contribute to more than one valve category). Highest early mortality occurred in active infective endocarditis, high-complexity (≥3-procedure), and non-elective operations, consistent with a high-acuity case-mix.

Table S5. Additional (non-significant) baseline characteristics.

Variable	Overall (*n*=821)	Non-survivors (*n*=187)	Survivors (*n*=634)	P
Body surface area, m²	1.8 [1.7–2.0]	1.8 [1.7–1.9]	1.8 [1.7–2.0]	0.39
Hypertension	329 (40.1)	82 (43.9)	247 (39.0)	0.24
Prior stroke	77 (9.4)	15 (8.0)	62 (9.8)	0.57
Preoperative atrial fibrillation	260 (31.7)	65 (34.8)	195 (30.8)	0.33
Outside-hospital referral	546 (66.5)	117 (62.6)	429 (67.7)	0.22

Data are median [interquartile range] or n (%); p from Mann–Whitney U or Fisher exact tests. None differed significantly between survivors and non-survivors.

Figure S1. Calibration of the preoperative risk model.

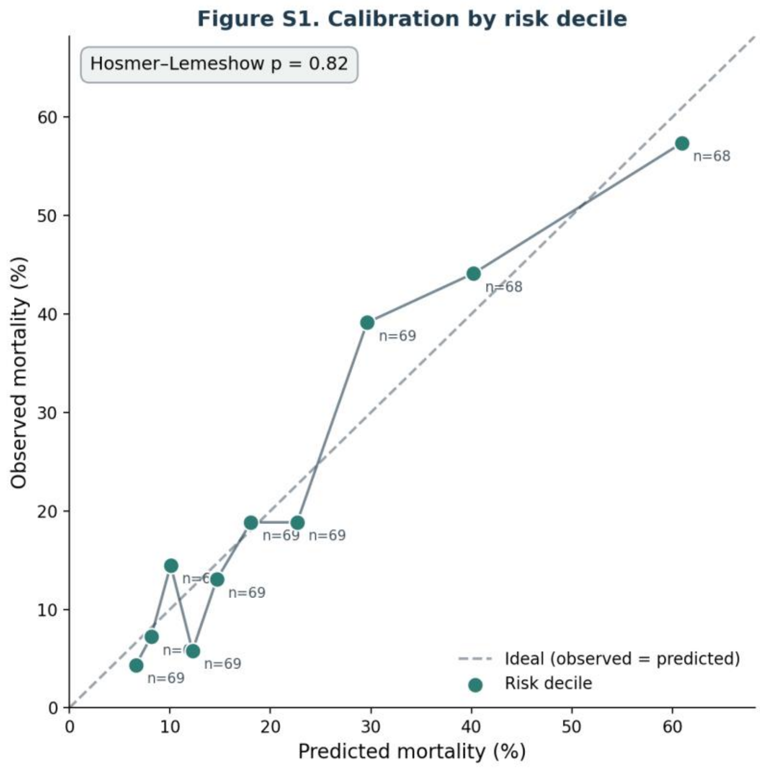

Figure S1. *Observed versus predicted early mortality by risk decile (n=685) lies close to the line of identity (Hosmer–Lemeshow p = 0.82), indicating good calibration. Marker size is proportional to decile size; the dashed line denotes perfect calibration.* Each decile contained 68–69 patients (highest-risk deciles D8–D10: *n*=69, 68, 68).

Figure S2. Patient flow through the study.

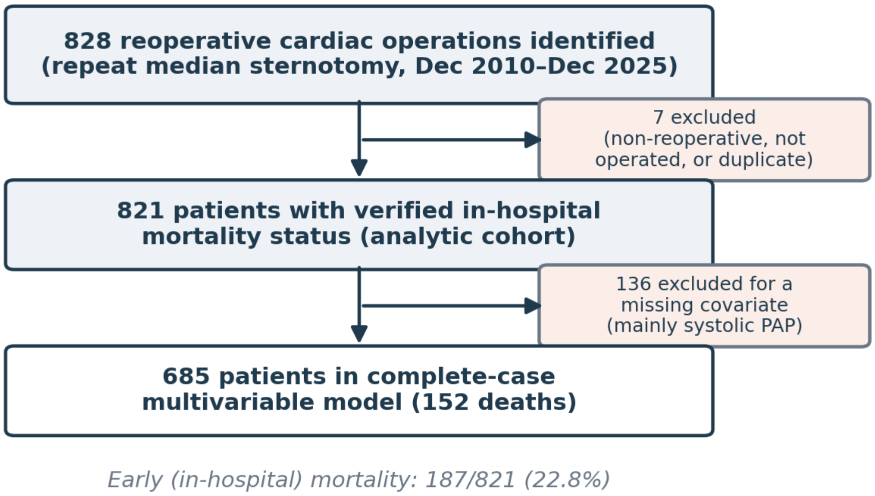

Figure S2. *Patient flow. Of 828 reoperative cardiac operations identified, 7 were excluded, leaving 821 with verified in-hospital mortality status; the complete-case multivariable model included 685 patients.*

Table S6. Sensitivity analysis: complete-case versus multiply-imputed (MICE) model for early mortality.

Predictor	Complete-case aOR (Table S1)	MICE-imputed aOR (*n*=821)
Chronic kidney disease	2.25	2.28
Age ≥70 years (vs <60)	2.32	2.44
Active endocarditis	1.89	2.06
NYHA III–IV	1.78	1.62
Non-elective status	1.69	1.46
Systolic PAP, per +10 mmHg	1.15	1.17
Model discrimination (AUC)	0.755	0.75

Complete-case column reproduces the primary model (Table S1). MICE performed on the full cohort (n=821) as a sensitivity analysis; all predictors retained their direction and magnitude, with non-elective status showing a borderline association. Discrimination is reported to two decimal places. aOR, adjusted odds ratio; AUC, area under the curve; MICE, multiple imputation by chained equations; PAP, pulmonary artery pressure.
